# Emulated trial investigating effects of multiple treatments: estimating combined effects of mucoactive nebulisers in cystic fibrosis using registry data

**DOI:** 10.1136/thorax-2023-220031

**Published:** 2023-07-14

**Authors:** Emily Granger, Gwyneth Davies, Ruth H Keogh

**Affiliations:** 1 Department of Medical Statistics, London School of Hygiene & Tropical Medicine, London, UK; 2 UCL Great Ormond Street Institute of Child Health, UCL, London, UK; 3 Respiratory Medicine, Great Ormond Street Hospital For Children NHS Foundation Trust, London, UK

**Keywords:** Cystic Fibrosis, Nebuliser therapy

## Abstract

**Introduction:**

People with cystic fibrosis (CF) are often on multiple long-term treatments, including mucoactive nebulisers. In the UK, the most common mucoactive nebuliser is dornase alfa (DNase). A common therapeutic approach for people already on DNase is to add hypertonic saline (HS). The effects of DNase and HS used alone have been studied in randomised trials, but their effects in combination have not. This study investigates whether, for people already prescribed DNase, adding HS has additional benefit for lung function or use of intravenous antibiotics.

**Methods:**

Using UK CF Registry data from 2007 to 2018, we emulated a target trial. We included people aged 6 years and over who were prescribed DNase without HS for 2 years. We investigated the effects of combinations of DNase and HS over 5 years of follow-up. Inverse-probability-of-treatment weighting was used to control confounding. The period predated triple combination CF transmembrane conductance regulator modulators in routine care.

**Results:**

4498 individuals were included. At baseline, average age and forced expiratory volume in 1 s (FEV_1_%) predicted were 21.1 years and 69.7 respectively. During first year of follow-up, 3799 individuals were prescribed DNase alone; 426 added HS; 57 switched to HS alone and 216 were prescribed neither. We found no evidence that adding HS improved FEV_1_% at 1–5 years, or use of intravenous antibiotics at 1–4 years, compared with DNase alone.

**Conclusion:**

For individuals with CF prescribed DNase, we found no evidence that adding HS had an effect on FEV_1_% or prescription of intravenous antibiotics. Our study illustrates the emulated target trial approach using CF Registry data.

**WHAT IS ALREADY KNOWN ON THIS TOPIC**

- People with cystic fibrosis (CF) are often prescribed multiple long-term treatments, including mucoactive nebulisers such as DNase and hypertonic saline. The effects of DNase and hypertonic saline used alone on health outcomes have been studied in randomised trials, but the combined effects of both treatments have not been studied.

**WHAT THIS STUDY ADDS**

- We emulated a hypothetical target trial using UK CF Registry data to compare multiple treatment strategies involving DNase and hypertonic saline. The primary interest was in estimating the effect of adding hypertonic saline to DNase after 2 years, compared with continued use of DNase alone, on long-term clinical health outcomes. We found no evidence that adding hypertonic saline has an effect on forced expiratory volume in 1 s or prescription of intravenous antibiotics.

**HOW THIS STUDY MIGHT AFFECT RESEARCH, PRACTICE OR POLICY**

- We provide an example of target trial emulation to answer a clinical question for which there is no evidence from randomised controlled trials. This approach may be used to address clinical questions that are unlikely to be answered in randomised trials in the field of CF and more widely.

## Background

Randomised controlled trials (RCTs) are the gold standard for evaluating the effects of treatments. However, there are many more questions relating to treatments than can reasonably be evaluated within RCTs. When there are multiple potential treatment strategies, for example, it can be challenging to recruit enough individuals in each treatment arm for sufficient power. An alternative is to use observational data to study treatment effects, and it is increasingly recognised that emulating a target trial when using observational data helps to clarify the research question and avoid common biases.^1–5^ In this study, we use registry data to emulate a target trial designed to compare multiple treatment strategies on health outcomes in people with cystic fibrosis (CF).

CF is an inherited condition caused by mutations in the CF transmembrane conductance regulator (CFTR) gene.^6^ This mutation leads to an abnormal movement of chloride and sodium across the airway epithelium. Thickened secretions and a cycle of inflammation and infection in the lungs result in significant morbidity.^6^ Many people with CF take mucoactive nebulisers which aim to alleviate the downstream consequences of CFTR dysfunction. The most commonly prescribed mucoactive nebuliser is dornase alfa (DNase),^7^ which is recommended as the first choice of mucoactive agent if there is clinical evidence of lung disease.^8^ DNase works by reducing viscosity in the lungs, which helps to clear lung secretions.^9^ In contrast, mucoactive nebulisers such as hypertonic saline (HS), help to clear lung secretions by rehydrating the airway surface liquid.^10^ Within clinical practice, depending on clinical status and clinician or patient preference, individuals already prescribed DNase may subsequently follow several different treatment strategies. These may include continuing on DNase alone, adding HS to their daily regimen, or, more rarely, a decision may be made to stop DNase and switch to HS.

RCTs have investigated the effects of DNase or HS alone in CF. A recent Cochrane systematic review^9^ found evidence to show that DNase alone may improve lung function and decrease pulmonary exacerbations in people with CF. Another Cochrane systematic review^10^ found evidence that HS alone can reduce pulmonary exacerbations and improve lung function, although the evidence for lung function was deemed low quality. Although DNase and HS are often prescribed in combination in clinical practice, their effects in combination have not been studied. Answering questions about the effects of different combinations of these two treatments using an RCT would be challenging, particularly if we are interested in studying long-term effects.

We use UK CF Registry data to emulate a target trial designed to compare multiple treatment strategies involving DNase and HS and assess their long-term effects on health outcomes in people with CF. The focus is on patients already established on DNase as defined by CF Registry documentation of current prescription, and the primary aim is to investigate the causal effect of adding and continuing HS versus continuing to use DNase only on two health outcomes: lung function (measured using forced expiratory volume in 1 s, FEV_1_%) and prescription of intravenous antibiotics. We compare outcomes measured at 1, 2, 3, 4 and 5 years of follow-up under each treatment strategy, where each treatment strategy is to be sustained up to the outcome measurement time. Previous studies have investigated the long-term effects of DNase using registry data and the findings suggest that DNase may improve the rate of decline in lung function^11 12^ and that it may be more beneficial for people with lower lung function.^13^ However, HS has not been previously studied using registry data either alone or in combination with DNase.

Our study is undertaken using data which predated the widespread introduction of triple combination CFTR modulator therapies into routine clinical care. The question we address is relevant to the CF population overall, although results from the premodulator period may not translate to a modulator-treated population. This will be able to be investigated using the same framework once more years of data are available. Furthermore, access to modulators is not universal globally, and in those countries with access, patients ineligible or unable to tolerate them represent an important minority in whom this question is particularly relevant.

There are several examples of using the target trial framework to compare treatment strategies across disease areas,^14–16^ including CF,^17^ however, they have tended to focus on treatment strategies involving a single treatment. In this study, we use this approach to compare treatment strategies that involve a combination of two treatments.

## Methods

### Study design and data source

Our study was designed to emulate a hypothetical RCT (ie, the ‘target trial’). The target trial framework involves describing the protocol for a randomised trial we would like to conduct if it were feasible, and then emulating that trial using the available observational data.^1^ A key element of the emulation of the trial involves controlling for confounding of the treatment-outcome association, as treatments are not randomly assigned in the observational data.

The key components of the protocol for our target trial are outlined in table 1. We emulate the target trial using data from the UK Cystic Fibrosis Registry.^18^ This is a national database managed by the Cystic Fibrosis Trust. Data are collected on time-invariant variables, such as sex, ethnicity and genotype, and variables that change over time. Longitudinal data are collected at approximately annual visits on over 250 variables covering several domains, including hospital admissions, pulmonary function, chronic medications, health complications, and these data have been recorded in a centralised database since 2007. For this study, data were available from 2007 to 2018. Further details on the registry are provided elsewhere.^18^

**Table 1:** Components of the target trial we aim to emulate in this study

Protocol component	Target trial	Emulation of the target trial using UK CF registry data
Eligibility criteria	Include: UK individuals with CF who have been taking DNase 2 years and are aged at least 6 years. Exclude: Individuals who have received an organ transplant, been treated with hypertonic saline within the last 2 years, or are taking mannitol, lumacaftor/ivacaftor or tezacaftor/ivacaftor	Include: Individuals observed in the UK CF Registry who meet the criteria in the target trial between 2007 and 2017, and who had at least 1 year of follow-up after baseline. Exclude: As in the target trial. We also exclude individuals with missing data on time-invariant confounders or FEV_1_% at baseline.
Treatment strategies	Continue DNase only and do not start hypertonic saline (DN) Continue DNase and add hypertonic saline immediately (DN&HS) Stop DNase and start hypertonic saline (HS) Stop DNase and do not start hypertonic saline (Nil) The treatment strategy is sustained for the duration of follow-up.	As in the target trial.
Assignment procedures	Participants will be randomly assigned to a treatment strategy when they are recruited to the trial. Participants and doctors will be aware of the treatment strategy they have been assigned to.	In the emulated trial individuals are not randomly assigned to the treatment strategy. This is accounted for in the analysis.
Follow-up period	1–5 years from randomisation.	1–5 years post-baseline.
Outcome	Lung function (measured using FEV_1_%) and use of intravenous antibiotics (yes/no)	As in the target trial.
Causal contrasts of interest	Per-protocol	As in the target trial.
Analysis plan	Estimate the mean difference in outcome between treatment strategies at follow-up for FEV_1_% and corresponding OR for use of intravenous antibiotics. Estimated using regression models for the outcome, with an indicator for treatment group and baseline measure of the outcome as explanatory variables.	Confounding by measured baseline and time-varying covariates is addressed using IPTW of MSM (see ‘Treatment effect estimands and statistical analysis’).

CF, cystic fibrosis; DNase, dornase alfa; FEV1%, forced expiratory volume in 1 s; IPTW, inverse-probability-of-treatment weighting; MSM, marginal structural model.

Table 1 specifies the components of the target trial and summarises how the registry data are used to emulate the target trial. Individuals meeting the inclusion criteria are those aged 6 years or older, who have been prescribed DNase but not HS for two consecutive years between 1 January 2007 and 31 December 2017. The baseline year is defined as the first year the inclusion criteria were met, so the earliest possible baseline year was 2008 and the latest was 2017. It is possible for individuals to meet the inclusion criteria in more than 1 year. When this was the case, we defined the baseline year to be the year from 2008 to 2017 which was most recent, but which allowed for the maximum possible follow-up time up to 5 years. For example, a person meeting the inclusion criteria in 2012 and 2013 has 6 and 5 years of potential follow-up, respectively, so we would choose 2013 as their baseline year. For a person meeting the inclusion criteria in 2013 and 2014, we would choose 2013. Exclusion criteria are listed in table 1.

Our treatments of interest are DNase and HS. At each annual review visit it is recorded whether individuals have been prescribed these treatments over the past year. Start and stop dates for long-term treatments are also available and these were used to impute any missing data on the treatments from the annual review visits. The two outcomes of interest are lung function and receipt of intravenous antibiotics. Both outcomes are recorded annually. Lung function is measured at annual visits by spirometry, with %-predicted values for FEV_1_% calculated using the Global Lung Initiative equations.^19^ At each annual visit the number of days on intravenous antibiotics (at home or in hospital) is recorded. In this study, use of intravenous antibiotics was treated as a binary variable indicating whether the individual has any recorded days on intravenous antibiotics since the last annual visit.

The emulation of the target trial uses existing observational data, reflecting data collected during routine clinical care and without any randomisation to treatment strategy. It is important that the analysis accounts for the lack of randomisation, as far as possible. In the observational data, the association between treatment and the outcome is suspected to be confounded by several factors. We used directed acyclic graphs to show the assumed relationships between the relevant variables in our data and to inform which variables should be considered as confounders (see online supplemental figures S.1 and S.2) in our analyses. The following variables were considered as potential confounders: sex, CF genotype, ethnicity, age, respiratory infections, intravenous hospital admissions, body mass index z-score, pancreatic insufficiency, use of CFTR modulators, past FEV_1_%, past intravenous antibiotic use and past rate of decline in FEV_1_%. Except for sex, genotype and ethnicity, which we take to be fixed over time, these covariates are recorded annually. Further details on how these covariates were defined are provided in online supplemental file.

10.1136/thorax-2023-220031.supp1 Supplementary data

### Treatment effect estimands and statistical analysis

The target trial specifies four longitudinal treatment strategies involving our two treatments of interest (table 1). Each treatment strategy involves beginning a particular combination of DNase and HS and sustaining that combination throughout follow-up. Our primary interest was in comparing the strategies of continuing DNase and adding HS (DN&HS) and continuing DNase only (DN). For the FEV_1_% outcome, the main estimands of interest were the mean differences in FEV_1_% at times 1–5 years had all individuals been following treatment strategy DN&HS, vs had all individuals been following treatment strategy DN. For intravenous antibiotics, the main estimands of interest were the corresponding ORs at times 1–4 years. Note that FEV_1_% is measured on the day of the annual review, whereas intravenous antibiotic use over the past year is recorded. To estimate 1-year, 2-year, 3-year, 4-year and 5- year treatment effects on FEV_1_%, we use FEV_1_% recorded at the first, second, third, fourth and fifth follow-up visit, respectively. To estimate the 1-year, 2-year, 3-year and 4- year treatment effects on intravenous antibiotic use, we use information recorded at the second, third, fourth and fifth follow-up visit. Comparisons between other treatment combinations were of secondary interest. We also compared the strategy of switching to and then continuing HS (HS) and the strategy of dropping DNase (Nil) with the strategy of continuing DNase only (DN).

The treatment effect estimands specified above were estimated using marginal structural models (MSM) estimated using inverse-probability-of-treatment weighting (IPTW).^20^ An MSM specifies how the outcome at a given time depends on treatment history up to that time, and in our case also on time and baseline covariates. The MSM cannot be fitted directly due to time-dependent confounding. IPTW involves estimating the probability of individuals receiving the treatment they received at each time point conditional on their treatment and covariate history up to that time. Multinomial regression was used to estimate the probability of having a given treatment combination (DN, DN&HS, HS, Nil). The IPTW at a given time is inverse of the product of the probabilities up to that time. Stabilised weights were used to avoid extreme weights.^21^

We assumed that consecutive visits were approximately 1 year apart. Some individuals had less than five follow-up visits after their baseline visit, due to the administrative end of follow-up, death or organ transplant. These individuals were censored at the time of death, transplant or end of follow-up. We did not use data from visits at which an individual had missing data in the outcome, or from visits at which individuals were using certain treatments (mannitol, lumacaftor/ivacaftor or tezacaftor/ivacaftor). Inverse-probability-of-censoring weights were used to address censoring, and inverse-probability-of-observation weights were used to handle exclusions at a given visit due to missing outcome data or use of certain treatments. Each individual had a combined weight at each time point which combines the IPTW with these other weights. Further details on the weights are provided in online supplemental file (see ‘Inverse-probability-of-treatment weighted estimation of marginal structural models’). As well as missing outcome data, there were missing data in some of the confounding variables. Full details on the amount of missing data and our approach to handling it are given in the online supplemental file (see ‘Missing data’). The MSM was fitted using the combined weights. For the FEV_1_% outcome the MSM is a linear regression model and for the intravenous antibiotic use outcome the MSM is a logistic regression model. The MSMs were fitted for all follow-up times combined with follow-up visit included as a covariate. The analysis for each outcome was conducted with and without interaction terms between treatment use and FEV_1_% at baseline in the MSM. The 95% CIs for the interaction terms were estimated to assess the evidence for treatment effect heterogeneity and we present treatment effects in individuals with low, moderate or high FEV_1_% at baseline by setting FEV_1_% to 40, 75 and 100, respectively. Full specification of the MSMs is provided in online supplemental file (see ‘Inverse-probability-of-treatment weighted estimation of marginal structural models’). All SEs and 95% CIs were estimated using the non-parametric bootstrap approach.

## Results

### Study population and descriptive statistics

**Figure 1:**
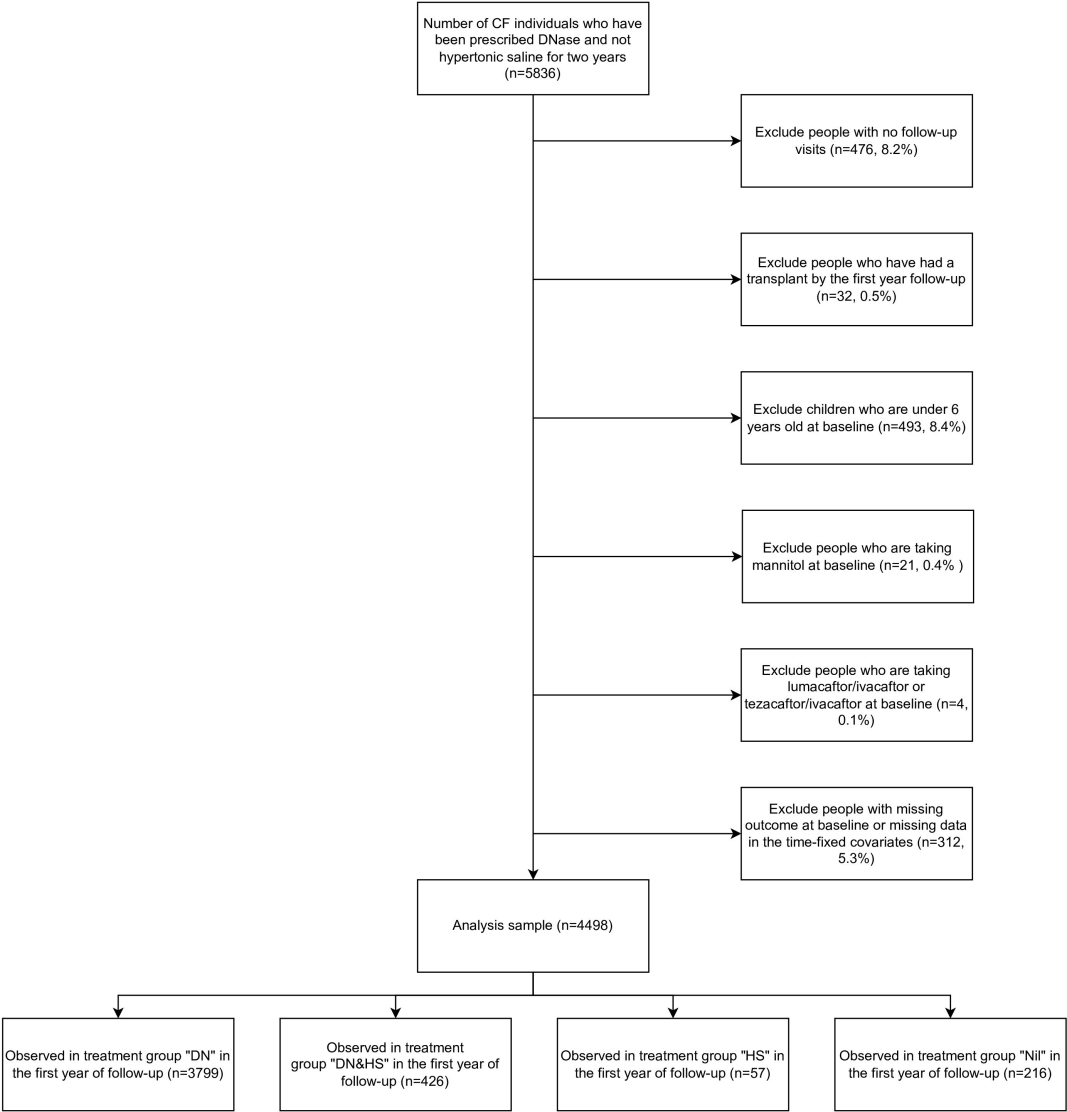
Flowchart of participant selection into the study sample. CF, cystic fibrosis; DNase, dornase alfa; DN&HS, DNase and adding hypertonic saline.

We identified 5836 individuals in the UK CF Registry who had been documented as having been prescribed DNase and not prescribed HS for at least two consecutive years between 2007 and 2017. Of these, 4498 individuals met our other inclusion and exclusion criteria for the emulated trial. Figure 1 describes the study sample derivation.

Table 2 summarises the characteristics, measured at baseline, of the study population, by the treatment combination they were observed to be using in the first year of follow-up. During the first year, 3799 individuals were prescribed DNase alone and 426 were prescribed both DNase and HS. Far fewer people were prescribed HS alone, or neither treatment (57 and 216, respectively). On average, individuals prescribed both treatments were the youngest (mean age: 18.7 years) and individuals taking neither treatment were the oldest (mean age: 24.2 years). Individuals prescribed HS alone had the lowest lung function (mean FEV_1_%: 66.2), whereas those prescribed DNase alone had the highest (mean FEV_1_% 70.1). The proportion of people who were recorded as taking no intravenous antibiotics in the year prior to baseline was highest for people prescribed neither treatment (46.3%) and lowest for individuals prescribed DNase and HS (35.4%). Individuals were observed to switch between treatment combination during the follow-up. Online supplemental figure S.3 shows the number of individuals prescribed each treatment combination by year, and online supplemental figure S.4 describes the flow of individuals between different treatment combinations by year. Of the 2521 individuals who were prescribed DNase alone in the first year and had 5 years of follow-up, 1615 (64.1%) remained on DNase alone for 5 years. Of the 260 individuals prescribed DNase and HS in the first year and who had 5 years of follow-up, 185 (71.2%) remained on DNase and HS for 5 years.

**Table 2:** Summary of characteristics at baseline overall and by treatment combination observed in the first year of follow-up

	DN (N=3799)	DN&HS (N=426)	HS (N=57)	Nil (N=216)	Whole cohort (N=4498)
Female, n (%)	1733 (45.6)	223 (52.3)	31 (54.4)	106 (49.1)	2093 (46.5)
Age, mean (SD)	21.3 (11.6)	18.7 (10.3)	19.7 (9.6)	24.2 (11.0)	21.1 (11.5)
Genotype risk group,* n (%)					
High	2994 (78.8)	358 (84.0)	46 (80.7)	154 (71.3)	3552 (79.0)
Low	301 (7.9)	24 (5.6)	3 (5.3)	22 (10.2)	350 (7.8)
None assigned	504 (13.3)	44 (10.3)	8 (14.0)	40 (18.5)	596 (13.3)
White ethnicity, n (%)	3653 (96.2)	409 (96.0)	56 (98.2)	208 (96.3)	4326 (96.2)
No of intravenous days over the past year, n (%)					
0	1665 (43.8)	151 (35.4)	24 (42.1)	100 (46.3)	1940 (43.1)
1–14	718 (18.9)	75 (17.6)	12 (21.1)	36 (16.7)	841 (18.7)
15–28	490 (12.9)	66 (15.5)	12 (21.1)	24 (11.1)	592 (13.2)
29+	926 (24.4)	134 (31.5)	9 (15.8)	56 (25.9)	1125 (25.0)
Intravenous hospital admissions,† n (%)	1638 (43.1)	220 (51.6)	28 (49.1)	91 (42.1)	1977 (44.0)
FEV_1_%, mean (SD)	70.1 (22.8)	67.9 (22.7)	66.2 (17.9)	66.6 (24.6)	69.7 (22.8)
Rate of decline in FEV_1_%, mean (SD)	−1.09 (1.50)	−1.33 (1.63)	−1.48 (1.28)	−1.23 (1.67)	−1.12 (1.52)
BMI z-score, mean (SD)	−0.05 (1.13)	−0.21 (1.13)	−0.31 (0.98)	−0.23 (1.26)	−0.08 (1.13)
*Pseudomonas aeruginosa* infection,‡ n(%)	2286 (60.2)	258 (60.6)	39 (68.4)	144 (66.7)	2727 (60.6)
*Staphylococcus* infection,‡ n (%)	1529 (40.2)	169 (39.7)	22 (38.6)	95 (44.0)	1815 (40.4)
Non-tuberculous, mycobacteria infection,‡ n (%)	173 (4.6)	25 (5.9)	4 (7.0)	11 (5.1)	213 (4.7)
Pancreatic insufficiency, n (%)	3367 (98.9)	392 (92.0)	48 (84.2)	186 (86.1)	3993 (88.8)
Prescribed ivacaftor, n (%)	42 (1.1)	4 (0.9)	0 (0.0)	4 (1.9)	50 (1.1)

Continuous variables are summarised using mean (SD) and categorical variables are summarised using numbers (%).

*High-risk and low-risk genotype classifications previously defined in Franklin *et al*.^22^ Genotypes which do not fall within either category were labelled ‘none assigned’.

†Intravenous hospital admissions: number of people with at least one intravenous hospital admission since the last review.

‡Infection data: indicator for any positive culture since the last review.

BMI, body mass index; DN, continue DNase and do not start hypertonic saline; DN&HS, continue DNase and start hypertonic saline; FEV1%, forced expiratory volume in 1 s; HS, drop DNase and start hypertonic saline; Nil, drop DNase and do not start hypertonic saline.

Details on the amount of missing data by follow-up year, and on the number of people who were censored each year (due to loss to follow-up, death or transplant), or temporarily excluded due to missing outcome data, are provided in online supplemental tables S.1 and S.2. Outcome trajectories by follow-up year and the distribution of weights used in the analysis are also provided in online supplemental figures S.5–S.7.

### Estimates of the effects of treatment combinations

**Figure 2:**
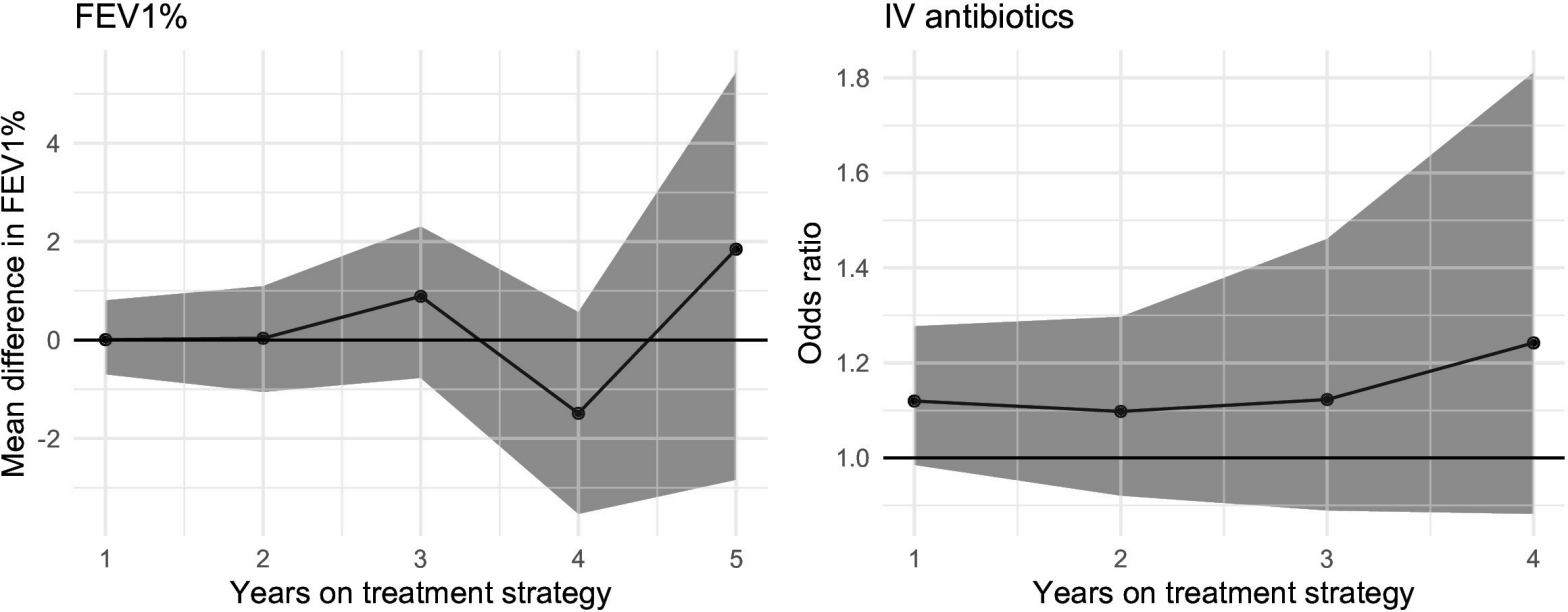
Estimated effects of adding hypertonic saline to DNase compared with continuing DNase alone on FEV_1_% and prescription of intravenous antibiotics. Mean differences are presented for FEV1% and ORs are presented for intravenous antibiotics. DNase, dornase alfa; FEV_1_%, forced expiratory volume in 1 s.

Figure 2 shows the expected mean differences in FEV_1_% (at times 1–5 years) and the odds of intravenous antibiotics versus no intravenous antibiotics (at times 1–4 years) between the two treatment strategies DN&HS vs DN (ie, the effect of adding HS on FEV_1_% and odds of intravenous antibiotics within an annual review year). Corresponding tabulated values are provided in online supplemental tables S.3 and S.4. For FEV_1_%, the mean differences are close to 0 at times 1–5, and all corresponding 95% CI contain 0. Similarly for intravenous antibiotics, the ORs are close to 1 at times 1–4, and all corresponding 95% CI contain 1. In other words, we found no evidence that adding HS would result in a different mean FEV_1_% or different odds of intravenous antibiotics among individuals who are already established on DNase, compared with continuing to use DNase only. For both outcomes, CIs, particularly for the later time points, were wide reflecting the uncertainty in our estimates.

Although not our focus, we also considered the effect of switching from DNase to HS (HS vs DN) and the effect of dropping DNase (Nil vs DNase). Figures for these additional comparisons are provided in the online supplemental figures S.8 and S.9.

### Treatment effects by baseline FEV_1_%

**Figure 3:**
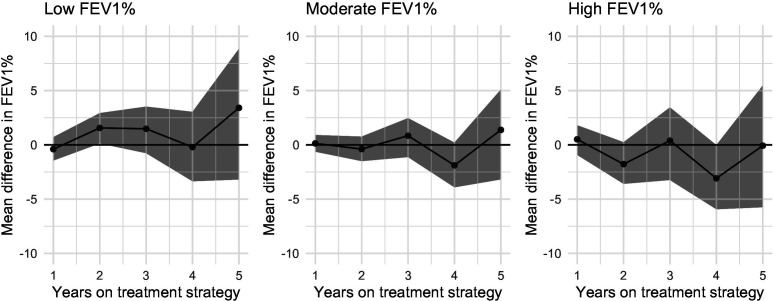
Estimated effects of adding hypertonic saline to DNase compared with continuing DNase alone on FEV1%. Estimated effects are mean differences and are presented for people with high (100) moderate (75) or low (40) FEV1% at baseline. DNase, dornase alfa; FEV1%, forced expiratory volume in 1 s.

**Figure 4:**
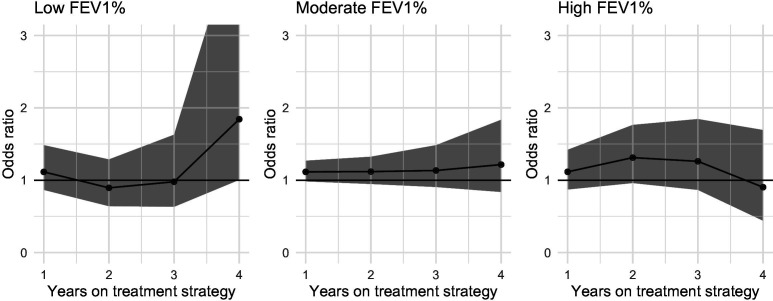
Estimated effects of adding hypertonic saline to DNase compared with continuing DNase alone on prescription of intravenous antibiotics. Estimated effects are ORs and are presented for people with high (100) moderate (75) or low (40) FEV1% at baseline. The upper limit of the 95% CI for the effect estimate at year 4 in the low FEV1% group is 4.55. DNase, dornase alfa; FEV1%, forced expiratory volume in 1 s.

We found no evidence of treatment effect heterogeneity by FEV_1_% at baseline. Figure 3 shows the expected mean differences in FEV_1_% at times 1–5 between DN&HS and DN, by FEV_1_% at baseline (low, moderate, high). Figure 4 shows the corresponding ORs at times 1–4.

For FEV_1_%, the estimated mean differences increase as baseline FEV_1_% decreases, suggesting that adding HS is more beneficial for individuals with lower FEV_1_%. However, the corresponding 95% CI all contain 0. Similarly, the results for the intravenous antibiotics outcome provide no evidence of an effect of adding HS to DNase at any level of FEV_1_%.

## Discussion

We used UK CF Registry data to emulate a hypothetical RCT designed to investigate the effects of multiple treatment strategies for mucoactive nebulised treatments on lung function and prescription of intravenous antibiotics in people with CF. Our primary interest was to investigate whether, for individuals already treated with DNase, adding HS has any additional benefit for these clinical health outcomes. We found no evidence of an effect (harmful or beneficial) of adding HS to DNase, either in change of FEV_1_% or prescription of intravenous antibiotics. We found some suggestion that adding HS may benefit lung function for people with lower initial FEV_1_%, although the results were not statistically significant. This is in line with results of a previous study based on UK CF Registry data, which suggested that the use of DNase alone could be more beneficial for FEV_1_% for people with lower initial FEV_1_%.^13^ People on both DNase and HS in the first year of follow-up tended to have lower lung function and more intravenous days, reflecting clinical practice where HS may be added to DNase when there has been clinical deterioration. This was addressed in the analysis by using IPTW to account for potential confounders.

Despite DNase and HS being commonly prescribed together in clinical practice, there have been no RCTs investigating the effects of these treatments used in combination. Hence, we demonstrate the application of target trial emulation to address a clinical question in CF for which there is no RCT evidence. The target trial framework applies the study design principles of RCTs to observational studies which helps to minimise biases that can arise due to study design and analysis choices.^1–5^ Target trial emulation has been used successfully in other disease areas to replicate the results from existing RCTs, for example, cardiovascular disease^22 23^ and diabetes.^24^ The UK CF Registry is cited as an exemplar patient registry in the NICE real-world evidence framework,^25^ holds pharmacovigilance credentials and hosts post-authorisation phase IV pharmacovigilance studies.^26^ It is the largest national CF registry outside of the USA and captures data on almost all the UK CF population.^18^ These data, coupled with target trial methodology, provide an opportunity for researchers in CF to address important questions for the CF community. We did not perform sample size calculations and there is some debate as to whether such calculations are needed in observational studies such as this.^27–29^ We used all the available data in the UK CF Registry, giving the biggest possible sample size for the study.

Although our primary interest was to investigate the effect of adding HS when established on DNase, we also investigated the effect of dropping DNase after 2 years and switching to HS after 2 years. Results suggested poorer outcomes in terms of FEV_1_% and intravenous antibiotics when dropping DNase, although results were largely non-significant. We found no evidence of an effect (in either direction) of switching to HS on intravenous days, although some evidence that switching to HS after 2 years can improve FEV_1_%. This result is not clinically plausible and may be impacted by unmeasured confounding, which we discuss further below. Switching to HS is a rare decision in clinical practice. The number of people prescribed HS alone reflects this (table 2).

There are several limitations to this study. A key limitation in analyses that use observational data to study treatment effects is the possibility of bias due to uncontrolled confounding. Our analyses crucially assume that we have captured all the reasons for prescribing different treatment combinations that are associated with the outcome. While our analyses have controlled for several factors considered as potential confounders, including indicators of disease severity, it is important to note the possibility of residual confounding due to factors we did not control for. There could be, for example, biological or socioeconomic factors^30^ that influence both the treatment strategy and the outcome but are not collected by the registry. A further limitation is that our analyses rely on accurate treatment data being entered into the registry by clinical teams. Recording of long-term treatments within the CF Registry captures whether the treatment has been prescribed over the past year, but there is no information on adherence to treatment or dosing regimen. It is, therefore, possible that some individuals did not take or were poorly adherent to their prescribed medicine, which could bias our results. Additionally, we were interested in the health outcomes FEV_1_% and pulmonary exacerbations (since previous trials have shown that DNase and HS can independently improve these outcomes), but we used intravenous antibiotic days as a proxy for exacerbations. The information on intravenous antibiotic days included both planned and unplanned intravenous and is not a direct marker of exacerbations. However, our approach is in line with previous studies using intravenous antibiotic days data from the UK CF Registry.^31 32^ Finally, the data used are from 2007 to 2018, and outcomes within the UK CF population are evolving rapidly with the introduction of CFTR modulators into routine care.^7^ This time frame, therefore, predates the widespread introduction of both dual and triple combination CFTR modulator therapy in the UK. It is unknown whether similar results would be obtained in patients established on highly effective modulator therapy. However, by predating the widespread introduction of CFTR modulators, we were, however, able to address this question without potential confounding by modulator status.

## Conclusions

In an emulated trial using observational UK CF Registry data, we saw no additional benefit to lung function or use of intravenous antibiotics when HS was added to DNase.

Our findings show that the UK CF Registry can support methodology for emulated trials. Although RCTs remain the gold standard, this methodology has the potential to address questions relevant to the CF community and could be particularly useful for assessing the long-term clinical effectiveness of multiple treatment strategies, since such questions are difficult to answer using a clinical trial. In future work, UK CF Registry data combined with the target trial framework could be used to repeat our study in a post-modulator population, including in groups with and without access to, or intolerant of, CFTR modulator treatments, and to answer related questions about discontinuing treatment in those using CFTR modulators.

## Data Availability

Data may be obtained from a third party and are not publicly available. To access the data, an application must be made to the UK CF Registry Research Committee. https://www.cysticfibrosis.org.uk/the-work-we-do/uk-cf-registry/apply-for-data-from-the-uk-cf-registry.
