# Supplementary material for: Emulated trial investigating effects of multiple treatments: estimating combined effects of mucoactive nebulisers in cystic fibrosis using registry data
Source: Thorax. 2023 Jul 14;78(10):1011–8. doi: 10.1136/thorax-2023-220031 (PMC10511967; doi:10.1136/thorax-2023-220031)
Supplement: Supplementary data [file thorax-2023-220031supp001.pdf]

SUPPLEMENTARY MATERIAL

AN EMULATED TRIAL INVESTIGATING THE EFFECTS OF MULTIPLE TREATMENTS: ESTIMATING COMBINED EFFECTS OF MUCOACTIVE TREATMENTS IN CYSTIC FIBROSIS USING REGISTRY DATA

Emily Granger[1], Gwyneth Davies[2], Ruth H. Keogh[1]

[1]Department of Medical Statistics,
Faculty of Epidemiology and Population Health,
London School of Hygiene and Tropical Medicine,
Keppel Street, London, WC1E 7HT

[2] Population, Policy and Practice Research and Teaching Department,
UCL Great Ormond Street Institute of Child Health (UCL GOS ICH),
London WC1N 1EH, United Kingdom

This document contains additional material on the methods (section 1) and results (section 2).

1. Additional notes on methodology

1.1 Causal estimands

The treatments of interest are dornase alfa and hypertonic saline. In the UK CF Registry, treatment use at each annual review is recorded as a yes/no indicating whether treatment was prescribed over the past year. Time is measured in years since baseline. Let $DN_{i,k}$ and $HS_{i,k}$ denote whether dornase alfa and hypertonic saline, respectively, was recorded for the *ith* person at time *k (k=1,2,3,4,5).* Let $A_{i,k}$ denote which treatment combination the *ith* person was on at time *k* (i.e. between times k-1 and k). Then $A_{i,k}$ is defined as:

$$A_{i,k} = \begin{cases} 0 \; if \; DN_{i,k} = 0 \; and \; HS_{i,k} = 0 \\ 1 \; if \; DN_{i,k} = 0 \; and \; HS_{i,k} = 1 \\ 2 \; if \; DN_{i,k} = 1 \; and \; HS_{i,k} = 0 \\ 3 \; if \; DN_{i,k} = 1 \; and \; HS_{i,k} = 1 \end{cases}$$

Henceforth we suppress the subscript i. The outcome at time k is denoted Y_k. The outcomes were FEV_1% (continuous, measured on the day of the annual review visit) and IV antibiotic use (binary, denoting whether or not any IV antibiotics were prescribed since the last annual review visit). Recall that at baseline (time 1) all individuals had been using DNase for 2 years, according to our inclusion criteria. Let $\bar{A}_k = \{A_1, \dots, A_k\}$ denote the treatment history from time 1 to time point k and let $Y_k^{\bar{a}_k}$ denote the potential outcome that would be observed for an individual with a particular treatment history $\bar{a}_k$. Our primary aim was to compare the strategies of adding hypertonic saline to DNase (denoted DN&HS) up to follow-up time of interest and continuing DNase alone up to the follow-up time of FEV_1% interest (denoted DN). Using our above notation, for the continuous outcome of the main estimands of interest are defined as:

1 year: $E\left(Y_1^{\bar{a}_1=3}\right) - E\left(Y_1^{\bar{a}_1=2}\right)$
2 year: $E\left(Y_2^{\bar{a}_2=(3,3)}\right) - E\left(Y_2^{\bar{a}_2=(2,2)}\right)$
3 year: $E\left(Y_3^{\bar{a}_3=(3,3,3)}\right) - E\left(Y_3^{\bar{a}_3=(2,2,2)}\right)$
4 year: $E\left(Y_4^{\bar{a}_4=(3,3,3,3)}\right) - E\left(Y_4^{\bar{a}_4=(2,2,2,2)}\right)$
5 year: $E\left(Y_5^{\bar{a}_5=(3,3,3,3,3)}\right) - E\left(Y_5^{a_5=(2,2,2,2,2)}\right)$

Comparisons between other treatment strategies were of secondary interest. We also compared the treatment strategies of switching from DNase to hypertonic saline and continuing HS alone to the follow-up time of interest (denoted HS) versus continuing DNase alone (denoted DN):

$$E\left(Y_k^{\bar{a}_k=\bar{1}}\right) - E\left(Y_k^{\bar{a}_k=\bar{2}}\right), k = 1, \dots, 5$$

We also compared the treatment strategies of dropping DNase and not adding hypertonic saline (denoted Nil) versus continuing DNase alone (DN):

$$E\left(Y_k^{\bar{a}_k=\bar{0}}\right) - E\left(Y_k^{\bar{a}_k=\bar{2}}\right), k = 1, \dots, 5$$

For the binary outcome of whether a person was prescribed any days of IV antibiotic treatment the estimands are odds ratios instead of mean differences. These are discussed in more detail in section 2.1.

1.2 Confounding variables

To obtain unbiased estimates of the treatment effects, we needed control for both time-invariant and time-varying confounders. Figures S.1 and S.2 are the directed acyclic graphs (DAGs) which show the assumed relationships between variables in our data for the analyses with FEV_1% and IV days as the outcome, respectively. Both DAGs are simplified versions of reality. We have not included long-

term arrows (e.g. from a variable recorded at time $k-2$ to one recorded at time k), or relationships between the time-dependent variables measured at a given visit. FEV_1% and BMI are recorded at the annual review visit. The following covariates (included together in a box in the DAGs) at a given annual visit refer to whether infection, pancreatic insufficiency, IV hospitalisation or ivacaftor prescription were recorded since the previous annual visit: pancreatic insufficiency, *Pseudomonas aeruginosa* infection, *Staphylococcus aureus* infection, *Nontuberculous Mycobacteria* infection, IV hospitalisation and Ivacaftor use.

Figure S. 1: Directed Acyclic Graph depicting the assumed short-term confounding paths of the treatment-outcome association when FEV_1% was the outcome (Y_i denotes FEV_1% at time i).

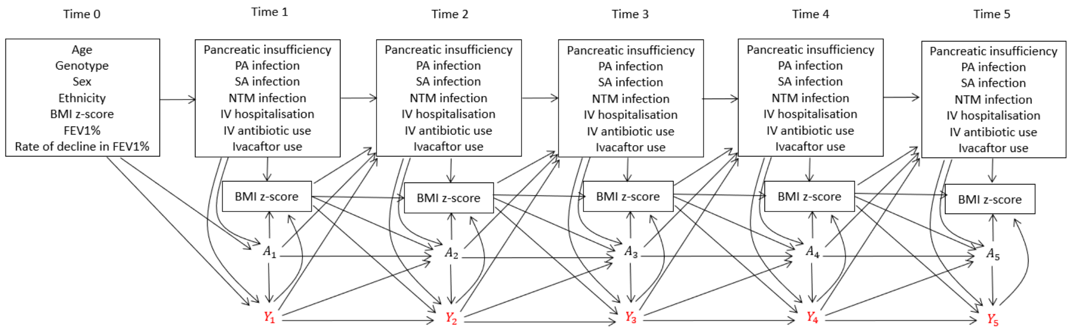

Figure S. 2: Directed Acyclic Graph depicting the assumed short-term confounding paths of the treatment-outcome association when IV days was the outcome (Y_i denotes binary indicator for IV prescription recorded at time i).

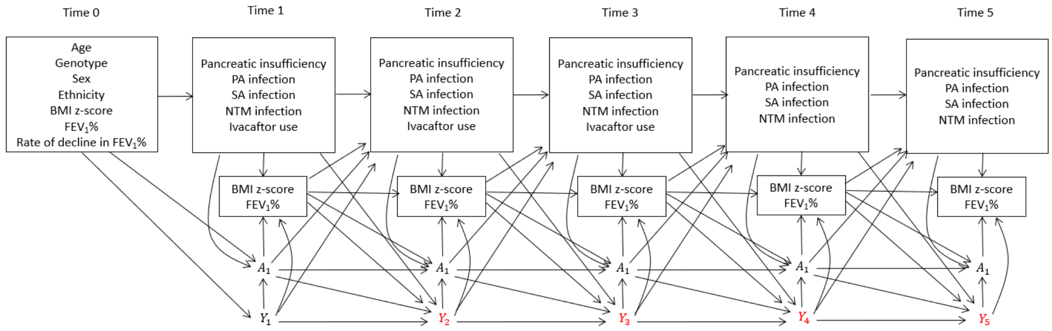

The subscripts denote follow-up year and subscript 0 denotes baseline. PA: Pseudomonas aeruginosa SA: Staphylococcus aureus; NTM: Nontuberculous Mycobacteria; IV: Intravenous antibiotics; BMI: Body Mass Index; A: Treatment combination.

As can be seen from Figures S.1 and S.2, the variables included as time-invariant confounders were: age at baseline, genotype, sex, ethnicity, rate of decline in FEV_1%, BMI z-score at baseline and FEV_1% at baseline. The variables included as time-varying confounders were: pancreatic insufficiency, ivacaftor use, *p. Aeruginosa* infection, *staphylococcus aureus* infection, *nontuberculous mycobacteria* infection, hospital admissions for intravenous antibiotics, days on intravenous antibiotics, past BMI z-score and past FEV_1%.

Genotype was classed as either high risk, low risk or not assigned as previously defined[1]. Ethnicity was classed as white or non-white due to small numbers in non-white ethnic groups in this population. Rate of decline in FEV_1% represented the change in FEV_1% observed prior to baseline. We defined the following linear mixed model with random slope and intercept:

$$FEV_1\%_{ij} = (\alpha_0 + \delta_{0i}) + (\alpha_1 + \delta_{1i})j + e_{ij}$$

Where $j \in \{0,1,2,3,4\}$ is the number of years before baseline ($j = 0$ is the baseline year). The estimate of the slope parameter ($\alpha_1 + \delta_{1i}$) for each individual is used as a time-invariant variable representing rate of change in FEV_1%.

Pancreatic insufficiency was a yes/no indicator where individuals were assigned "yes" if they were prescribed pancreatic enzyme supplements. IV hospital admissions was a yes/no indicator were yes indicated individuals had at least one hospital admission for IV antibiotics over the past year. IV days included home and hospital admissions and was categorised as: 0, 1-4, 15-28 and 29+. BMI z-scores were calculated using the WHO reference distribution[2] and FEV_1% was calculated using the Global Lung Initiative equations[3].

1.3 Inverse-probability-of-treatment weighted estimation of marginal structural models.

Let $\boldsymbol{L_B}$ denote the set of time-invariant confounders and $\boldsymbol{L_k}$ denote the set of time-varying confounders recorded at time *k.* In both the FEV_1% and IV days analyses,

$$\boldsymbol{L_B} = \{Age_0, Genotype, Sex, Ethnicity, Rate\ of\ decline\ in\ FEV1\%, FEV1\%_0 BMI_0\}$$

For the FEV_1% analysis, $\boldsymbol{L_k}$ is defined as:

$$\boldsymbol{L_k} = \{FEV1\%_{k-1}, BMI_{k-1}, IV\ days_k, IV\ Hospital\ Admission_k,$$

$$NTM_k, SA\ infection_k, PA\ infection_k, Ivacaftor\ use_k, Pancreatic\ insufficiency_k\}$$

For the IV days analysis, $\boldsymbol{L_k}$ is defined as:

$$\boldsymbol{L_k} = \{FEV1\%_{k-1}, BMI_{k-1}, IV\ days_k,$$

$$NTM_k, SA\ infection_k, PA\ infection_k, Ivacaftor\ use_k, Pancreatic\ insufficiency_k\}$$

Then, the stabilised inverse-probability-of-treatment weights for individual i at time k ($IPT.w_{ik}$) were defined as:

$$IPT.w_{ik} = \frac{\prod_{j=0}^{k} \Pr\left(A_j = a_{ij} \middle| \bar{A}_{j-1} = \bar{a}_{ij-1}, \boldsymbol{L_B} = \boldsymbol{l_i}\right)}{\prod_{j=0}^{k} \Pr\left(A_j = a_{ij} \middle| \bar{A}_{j-1} = \bar{a}_{ij-1}, \boldsymbol{L_B} = \boldsymbol{l_i}, \boldsymbol{\bar{L}_j} = \boldsymbol{\bar{l}_{ij}}\right)}$$

Weights were also used to account for missing data in FEV_1% or BMI ($MISS.w_{ik}$), loss-to-follow-up ($LTFU.w_{ik}$), censoring due to death or transplant ($CENS.w_{ik}$) and time-varying eligibility due to use of lumacaftor/ivacaftor or texacaftor/ivacaftor ($LUTE.w_{ik}$) or mannitol ($MANN.w_{ik}$).

The probabilities required for each set of weights were obtained using logistic regression. For $LUTE.w_{ik}$, and $MANN.w_{ik}$, the outcomes were indicators for use of the relevant treatments.

For each individual, we excluded time-points with missing data for FEV_1% or BMI. To account for the missing data, the remaining individuals were re-weighted by the inverse of their probability of remaining in the study at a given time. The weights, $MISS.w_{ik}$, were defined using a similar equation as the one for $IPT.w_{ik}$, but the outcome was an indicator for missingness in FEV_1% or BMI for the ith individual at time k.

Individuals who were lost to follow-up, died or had an organ transplant were censored at the time of the event. For the loss to follow-up weights, the outcome at time *k* was an indicator for whether the individual was lost to follow-up at time $k + 1$. For the censoring weights due to death or organ transplant (whichever occurred first), the outcome at time *k* was an indicator for whether the individual died or had a transplant between times k and $k + 1$.

All weights were stabilised and probabilities were conditioned on the same variables as the probabilities defined in the inverse-probability-of-treatment weights.

The combined weight for individual i at time point k ($COMBINED.w_{ik}$) was defined as a product of all of the above weights:

$$COMBINED.w_{ik} = IPT.w_{ik} \times LUTE.w_{ik} \times MANN.w_{ik} \times MISS.w_{ik} \times CENS.w_{ik} \times LTFU.w_{ik}$$

For our main analysis, we specified the following linear marginal structural model (MSM) for the continuous outcome of FEV$_1$%:

$$Y_{ik}^{\bar{a}_k} = \beta_0 + \sum_{j=1}^{k}\sum_{c=1}^{3} \boldsymbol{\beta_{cj} I(a_j = c)} + \boldsymbol{\beta_B L_{Bi}} + \beta_k k + \varepsilon_{ik}, k = 1, \dots, 5$$

The parameters of the MSM are estimated by fitting the model using the observed data weighted using the combined weight. This enables estimation of the estimands specified in supplementary section 1.1. We note that these are marginal mean differences, as the conditional and marginal mean differences coincide for the linear MSM above. .

For the binary outcome of whether the individual was prescribed any IV antibiotics over the past year, the marginal structural model (MSM) used for the main analysis was:

$$\log\left(\frac{\Pr\,(Y_{ik+1}^{\bar{a}_k} = 1|L_{Bi})}{\Pr\,(Y_{ik+1}^{\bar{a}_k} = 0|L_{Bi})}\right) = \beta_0 + \sum_{j=1}^{k}\sum_{c=1}^{3} \boldsymbol{\beta_{cj} I(a_j = c)} + \boldsymbol{\beta_B L_{Bi}} + \beta_k(k+1) + \varepsilon_{ik}, k = 1, \dots, 4$$

This can be fitted using the observed data weighted using the combined weight. This results in estimates of conditional odds ratios. For example, our primary odds ratios of interest are

$$OR_k^{DN\&HS\ vs\ DN} = \frac{\Pr\left(Y_{k+1}^{\bar{a}_k=3} = 1|\boldsymbol{L}_B\right)/\Pr\,(Y_{k+1}^{\bar{a}_k=3} = 0|\boldsymbol{L}_B)}{\Pr\left(Y_{k+1}^{\bar{a}_k=2} = 1|\boldsymbol{L}_B\right)/\Pr\,(Y_{k+1}^{\bar{a}_k=2} = 0|\boldsymbol{L}_B)}, k = 1, \dots, 4$$

For the analyses investigating whether the treatment effects differed by FEV$_1$% measured at baseline, the above MSMs were extended to include an interaction between FEV$_1$% (a component of L_B) and $I(a_j = c)$.

2. Additional results

2.1 Missing data

We found 5360 individuals with CF who were documented as having been prescribed dornase alfa and not hypertonic saline for at least two consecutive years between 2007 and 2017, and who had at least one baseline visit and one follow-up year. After excluding individuals who were under the age of 6 years, had received a solid organ transplant by baseline, or were prescribed mannitol, tezacaftor/ivacaftor or lumacaftor/ivacaftor at baseline, we were left with 4810 individuals who were eligible for inclusion. Table S.1 shows the amount of missing data by year for those individuals. Note that this includes people who were transplanted, or prescribed mannitol, tezacaftor/ivacaftor or lumacaftor/ivacaftor post-baseline.

Table S.1: Amount of missing data in the 4810 individuals eligible for inclusion in the study, by year

Year (k)	0 (n=4810)	1 (n=4810)	2 (n=4471)	3 (n=4078)	4 (n=3660)	5 (n=3261)
Treatment strategy	0 (0%)	0 (0%)	0 (0%)	0 (0%)	0 (0%)	0 (0%)
FEV$_1$%	235 (4.9%)	220 (4.6%)	175 (3.9%)	149 (3.7%)	164 (4.5%)	131 (4.0%)
Number of IV days	0 (0%)	0 (0%)	0 (0%)	0 (0%)	0 (0%)	0 (0%)
Sex	0 (0%)	0 (0%)	0 (0%)	0 (0%)	0 (0%)	0 (0%)
Genotype	44 (0.9%)	44 (0.9%)	44 (0.9%)	44 (0.9%)	44 (0.9%)	44 (0.9%)
Ethnicity	33 (0.7%)	33 (0.7%)	33 (0.7%)	33 (0.7%)	33 (0.7%)	33 (0.7%)
Age	0 (0%)	0 (0%)	0 (0%)	0 (0%)	0 (0%)	0 (0%)
Rate of decline in FEV$_1$%	0 (0%)	0 (0%)	0 (0%)	0 (0%)	0 (0%)	0 (0%)
Ivacaftor use	0 (0%)	0 (0%)	0 (0%)	0 (0%)	0 (0%)	0 (0%)
P. aeruginosa infection	0 (0%)	3 (0.1%)	10 (0.2%)	13 (0.3%)	6 (0.2%)	5 (0.2%)
Staphylococcus aureus infection	0 (0%)	3 (0.1%)	10 (0.2%)	13 (0.3%)	6 (0.2%)	5 (0.2%)
NTM	0 (0%)	3 (0.1%)	11 (0.2%)	13 (0.3%)	7 (0.2%)	7 (0.2%)
IV hospital admission	0 (0%)	0 (0%)	0 (0%)	0 (0%)	0 (0%)	0 (0%)
Lagged BMI z-score*	-	79 (1.6%)	79 (1.6%)	85 (1.9%)	57 (1.4%)	59 (1.6%)

Pancreatic Insufficiency	0 (0%)	0 (0%)	0 (0%)	0 (0%)	0 (0%)	0 (0%)

**Lagged BMI z-score at visit i refers to the BMI z-score at visit k-1*

We excluded individuals with missing data on time-invariant variables (genotype and ethnicity) and individuals with missing FEV_1% data at baseline ($k = 0$).

The last observation carried forward was used to impute the missing infection data (*P. aeruginosa* infection, *Staphylococcus aureus* infection, *Bukholderia cepa* infection and NTM). This was considered a valid approach as these infections are usually long-term and there was no missing data in these variables at time 0.

Missing data weights were used to account for missing BMI and FEV_1% (except for individuals who had missing FEV_1% at baseline, who were excluded).

2.2 Summary of exclusions due to loss to follow-up, death, transplant, ineligibility and missing data.

Table S.2 gives the numbers of individuals who were excluded or censored each year for different reasons. Individuals who were censored due to loss-to-follow-up, death or transplant, and these individuals account for the decreasing number observed by follow-up year. For example, in between visits 1 and 2, 250 individuals were lost-to-follow-up, 58 died and 29 received an organ transplant. By visit 2, 4498-(250+58+29)=4161 individuals remained in the study.

Individuals who were temporarily excluded due to missing data or temporary ineligibility (due to initiating treatment with CFTR modulators or mannitol) were allowed to re-enter the study, and these numbers account for the differences between the number of people observed in each follow up year and the number of people included in the final analysis (final N).

Column percentages are given with respect to the sample sizes in row 1.

Table S.2: Number of people censored for different reasons by year

Follow-up year:	1	2	3	4	5
Number observed*	4498	4161	3776	3365	2975
LTFU**	0 (0%)	250 (5.6%)	303 (7.3%)	305 (8.1%)	286 (8.5%)
Death**	0 (0%)	58 (1.3%)	57 (1.4%)	79 (2.1%)	74 (2.2%)
Transplant**	0 (0%)	29 (0.6%)	25 (0.6%)	27 (0.7%)	30 (0.9%)
Mannitol	20 (0.4%)	42 (1.0%)	56 (1.5%)	88 (2.6%)	120 (4.0%)
Prescribed CFTR modulators	8 (0.2%)	15 (0.4%)	13 (0.3%)	14 (0.4%)	35 (1.2%)
Missing data	162 (3.6%)	257 (6.2%)	222 (5.9%)	197 (5.9%)	186 (6.3%)
Final N***	4308	3847	3485	3066	2634

LTFU: Lost to follow-up; CFTR modulators: these include lumacaftor/ivacaftor and tezacaftor/ivacaftor; Missing data: this is missing data in FEV_1% or BMI z-score.
**Number observed: this gives the number of individuals who remained in the study by visit k.*
***Numbers for visit k denote individuals who were lost-to-follow-up, died or received a transplant between visits k and k+1.*
****Final N: final number of people included in the analysis each year after censoring and temporary exclusions.*

2.3 Summary of the numbers of people prescribed each treatment combination and flow of participants between treatment combinations by year

Figure S.3 shows the number of people prescribed each treatment combination by year. Across all follow-up years, the percent of individuals using neither DNase nor hypertonic saline ranged between 4.2% and 5.2%. The percentages prescribed DNase only and hypertonic saline only ranged from 51.7%-81.2% and 1.2%-2.8% respectively. The percentage prescribed both DNase and hypertonic saline ranged from 9.1%-29.6%.

Figure S.4 describes the flow of participants between treatment combinations by year. Of the 143 individuals who were using neither DNase nor hypertonic salin in the first year and had 5 years of follow-up, 31 (21.7%) continued to use neither treatment for 5 years. Of the 51 individuals using hypertonic saline only in the first year (i.e, who switched from DNase to hypertonic saline) and had 5 years of follow-up, 16 (31.4%) remained on hypertonic saline only for 5 years. Of the 2521 individuals who continued to be prescribed DNase only in the first year and had 5 years of follow-up, 1615 (64.1%) remained on DNase only for 5 years. Of the 260 individuals who added hypertonic saline to DNase in the first year and had 5 years of follow-up, 185 (71.2%) remained on this combination for 5 years.

Figure S.3: Flowchart showing the number of participants in the study and number of participants prescribed each treatment combination by year.

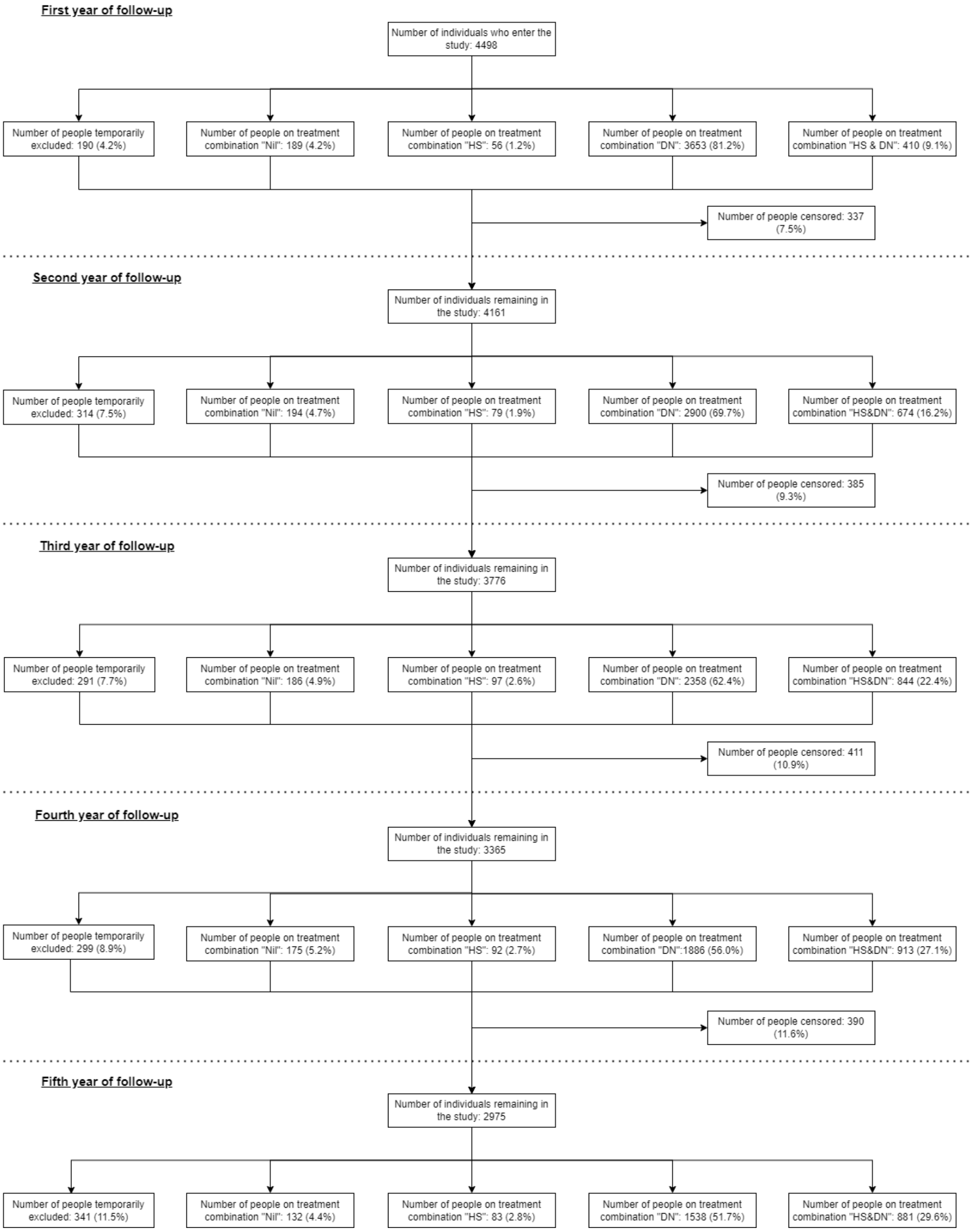

The percentages given in the first follow-up year are percentages of the total number of individuals who entered the study. The percentages given in follow-up years 2-5 are percentages of the number of individuals who remained in the study in follow-up years 2-5, respectively.

Figure S.4: Flow of participants prescribed each treatment combination by follow-up year

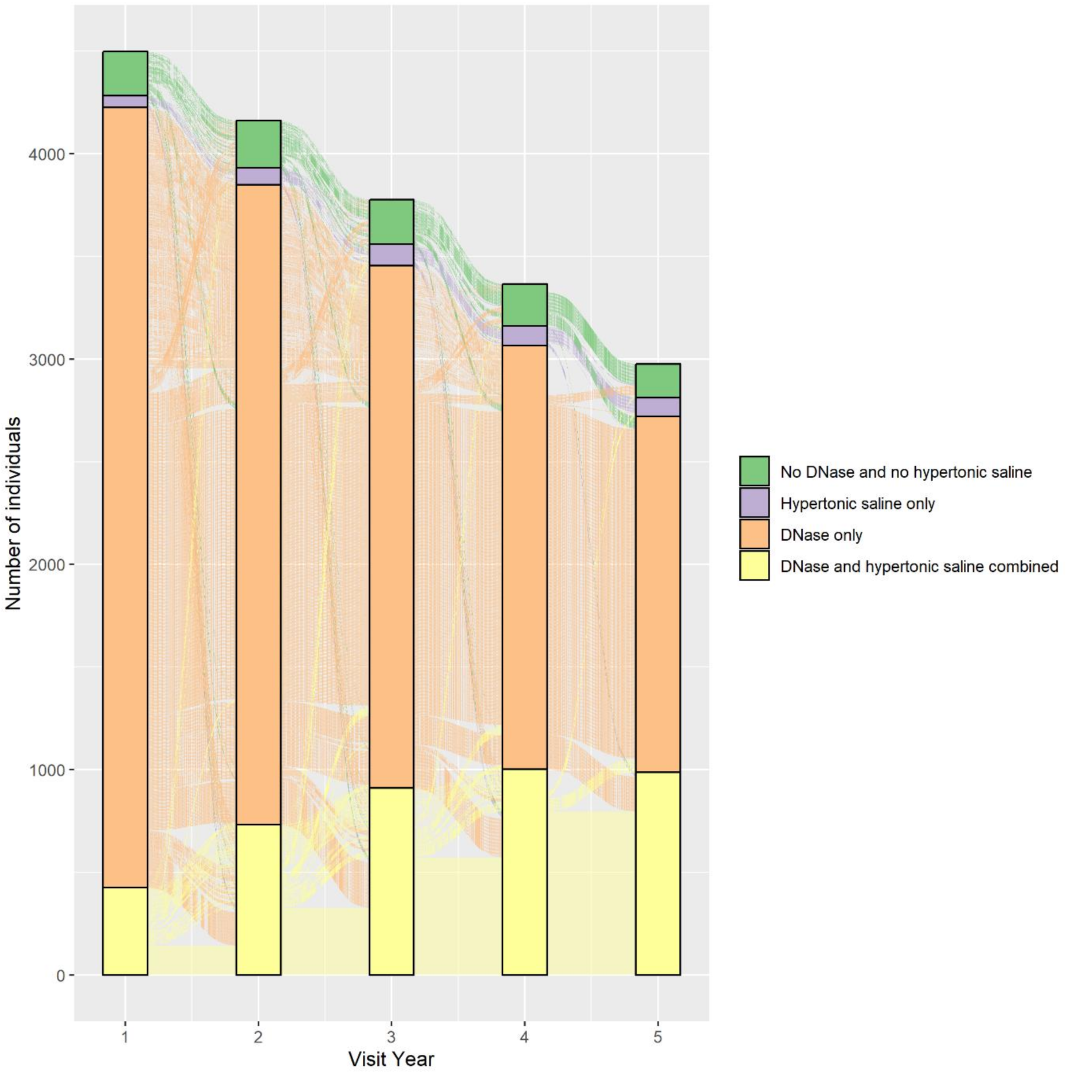

2.3 Outcome trajectories in the whole cohort

Figure S.5 shows the average FEV_1% and the proportion of individuals with at least one day on IV antibiotics in the whole cohort, by follow-up visit. The average FEV_1% decreases by year, whereas the proportion of individuals on IV antibiotics increases by year.

Figure S. 5: Average FEV_1% and proportion of people on IV antibiotics in the whole cohort, by follow-up visit. Note that the vertical axes are truncated and the changes over time are small.

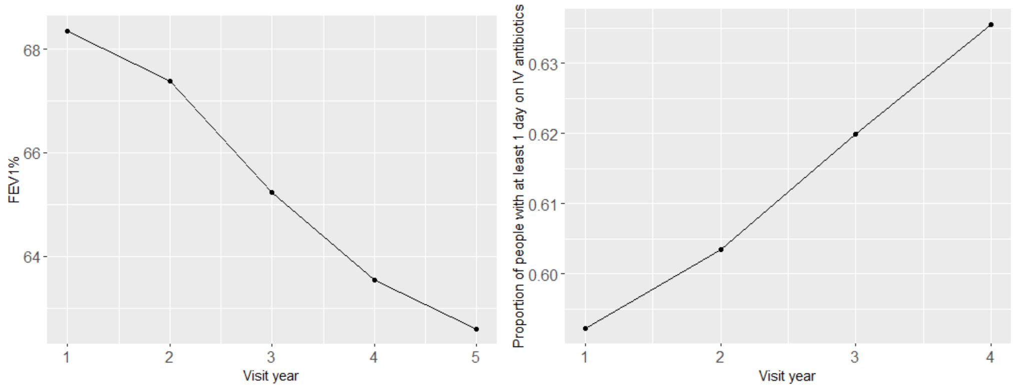

2.4 Distribution of weights

Figures S.6 and S.7 show the distribution of inverse-probability-of-treatment (IPT) weights and combined weights by year, respectively (weights are defined in Section 1.3). Boxplots show that the median weights are approximately 1 for each year, as expected. The variance of weights tends to increase by year but there are no extreme values.

Figure S. 6: Distribution of inverse-probability-of-treatment (IPT) weights by year. Horizontal line at y=1.

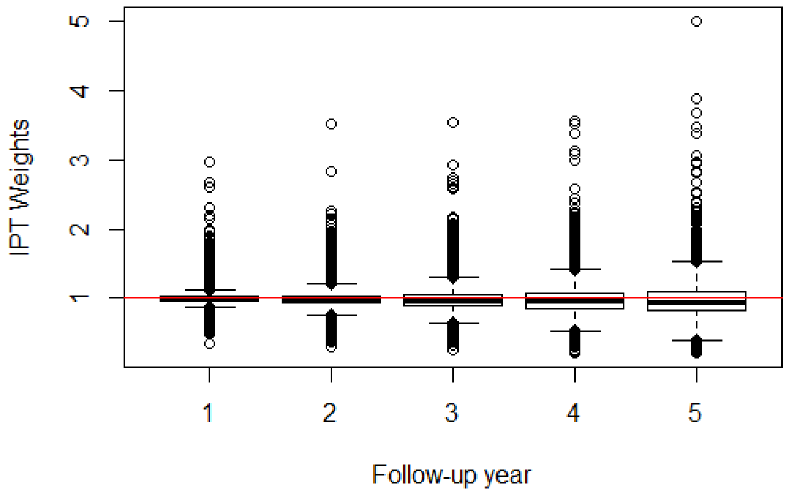

Figure S. 7: Distribution of combined weights by year. Horizontal line at y=1.

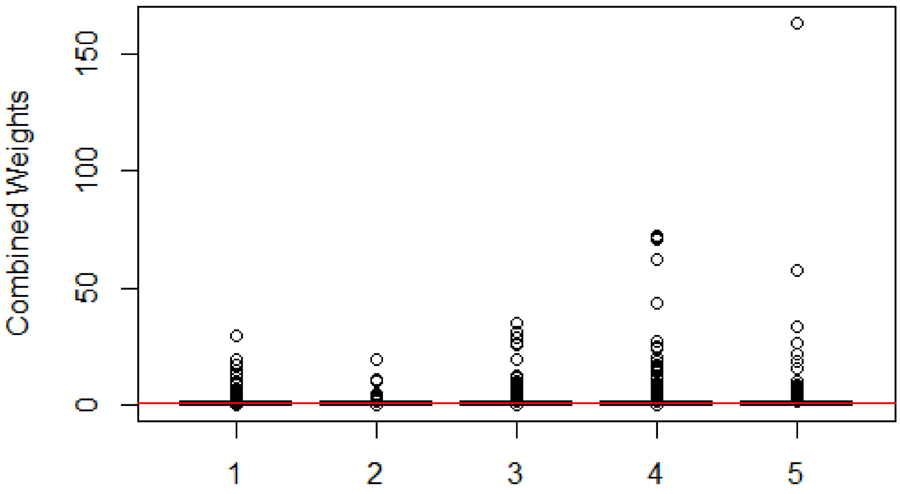

2.5 Comparing different treatment strategies

Figure S.8 shows the expected mean differences in FEV_1% at times 1-5 years between the following treatment strategies: (1) DN&HS versus DN; (2) HS versus DN and (3) Nil versus DN. The three comparisons can be interpreted as follows: (1) the effect of adding hypertonic saline (2) the effect of switching from DNase to hypertonic saline and (3) the effect of stopping DNase. Figure S.9 shows the estimated odds ratios for IV antibiotic treatment at times 1-4 years in the 'active' treatment strategies DN & HS, HS, Nil versus the comparator treatment strategy DN. Odds ratios above 1 indicate a larger odds of having IV therapy in the active treatment strategy.

The results show evidence of a beneficial effect of switching to hypertonic saline in terms of FEV_1%, but no effect (beneficial or harmful) on IV antibiotic use. Estimated effects of stopping DNase are negative for FEV_1% and positive for IV antibiotic use (indicating worse outcomes in people who stop DNase in both cases). However, there is no evidence of an effect on either outcome, with all 95% confidence intervals containing the null value (0 for FEV_1% and 1 for IV antibiotic use).

Figure S.8: Estimated effects (and 95% CIs) for multiple treatment strategy comparisons, for 1-5 years, on FEV_1%

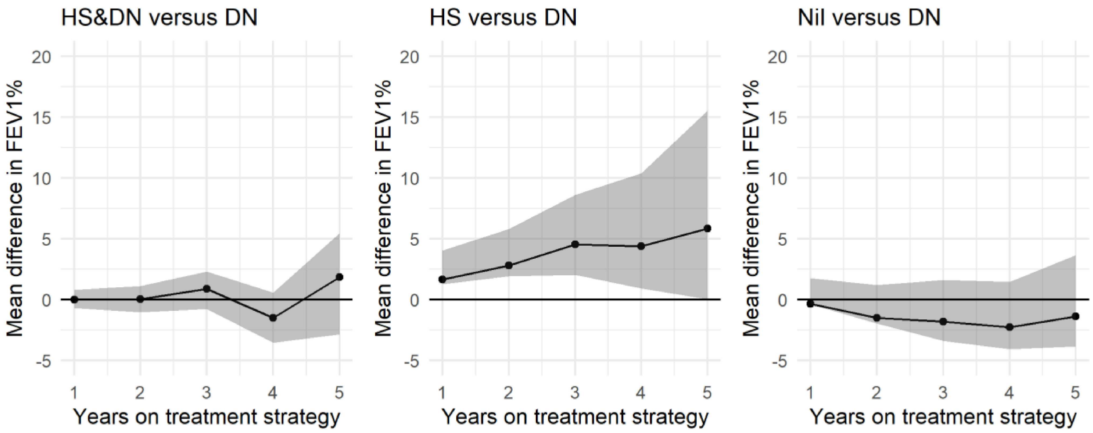

Figure S.9: Estimated effects (and 95% CIs) for multiple treatment strategy comparisons, for 1-4 years, on odds of being prescribed IV antibiotics

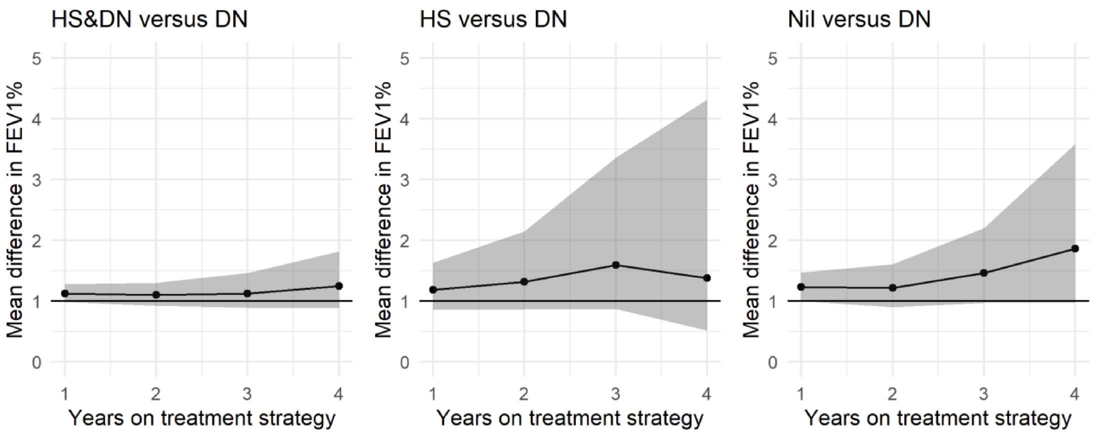

2.7 Tabulated results

Table S.3 presents the estimated mean differences in FEV_1%, at times 1-5, between the two treatment strategies DN&HS versus DN, for the whole cohort, and conditional on having high, medium or low FEV_1% at baseline. High medium and low are defined as 100, 75 and 45, respectively. Table S.4 presents the odds of IV antibiotics versus no IV antibiotics, at times 1-4, between the two treatment strategies DN&HS versus DN, for the whole cohort, and conditional on having high, medium or low FEV_1% at baseline.

Table S.3: Estimated mean difference in FEV_1% comparing HS&DN versus DN for 1-5 years for the whole cohort, and conditional on high (100), moderate (75) or low (45) FEV_1% at baseline

Year	Whole cohort	High FEV_1%	Moderate FEV_1%	Low FEV_1%
1	0.01 (-0.70, 0.81)	0.51 (-0.93, 1.81)	0.13 (-0.63, 0.91)	-0.40 (-1.44, 0.71)
2	0.04 (-1.05, 1.09)	-1.78 (-3.59, 0.25)	-0.39 (-1.50, 0.76)	1.56 (0.13, 2.93)
3	0.89 (-0.77, 2.31)	0.39 (-3.24, 3.45)	0.85 (-1.13, 2.46)	1.48 (-0.76, 3.53)
4	-1.49 (-3.53, 0.57)	-3.09 (-5.93, -0.02)	-1.89 (-3.91, 0.21)	-0.21 (-3.36, 3.05)
5	1.85 (-2.84, 5.44)	-0.07 (-5.75, 5.47)	1.38 (-3.18, 5.10)	3.41 (-3.19, 8.85)

Table S.4: Estimated odds ratios of HS&DN versus DN for 1-5 years on prescription of IV antibiotics for the whole cohort, and conditional on high (100), moderate (75) or low (45) FEV_1% at baseline

Year	Whole cohort	High FEV_1%	Moderate FEV_1%	Low FEV_1%
1	1.12 (0.99, 1.28)	1.12 (0.87, 1.42)	1.12 (0.98, 1.27)	1.11 (0.87, 1.48)
2	1.10 (0.92, 1.30)	1.31 (0.96, 1.76)	1.12 (0.95, 1.32)	0.89 (0.64, 1.29)
3	1.12 (0.89, 1.46)	1.26 (0.87, 1.85)	1.13 (0.91, 1.48)	0.98 (0.63, 1.63)
4	1.24 (0.88, 1.81)	0.90 (0.44, 1.69)	1.22 (0.84, 1.84)	1.84 (1.01, 4.55)

References

1. McKone E, Goss C, Aitken M. CFTR Genotype as a Predictor of Prognosis in Cystic Fibrosis. *Chest* 2006; 130: 1141–1147.

2. World Health Organisation. BMI-for-age (5-19 years). 2021. Available at: www.who.int. https://www.who.int/toolkits/growth-reference-data-for-5to19-years/indicators/bmi-for-age. [Accessed September 14, 2021]

3. Hall GL, Stanojevic S, Executive GLIN, Members of the GLINE. The Global Lung Function Initiative (GLI) Network ERS Clinical Research Collaboration: how international collaboration can shape clinical practice. *Eur Respir J*. 2019;53(2). doi:10.1183/13993003.02277-2018
